# Building Narratives of Effectiveness and Customer Orientation at the Interface of Different Actors in the Lifestyle Guidance Service Process

**DOI:** 10.1177/23743735241312802

**Published:** 2025-01-15

**Authors:** Heli Tiusanen, Sanna Ryynänen, Marjo Suhonen

**Affiliations:** 1 Faculty of Social Sciences, The Center of Expertise on Social Welfare in South-East Finland (Socom), Lappeenranta, Finland; 2 Department of Administrative Sciences, University of Lapland, Rovaniemi, Finland

**Keywords:** lifestyle counselling, effectiveness, customer orientation, service process

## Abstract

Co-production is expected to lead to more efficient and effective services. In response, this study aims to describe and understand the construction of effectiveness and the manifestation of customer orientation among the actors involved in the service process of lifestyle guidance. A qualitative narrative study was designed, and data were collected through the thematic interviews (n = 9) with the management, employees and customers of a Finnish public healthcare and social welfare organisation. The main narratives were as follows: (1) Shared value creation narrative, (2) Organisational strategy and customer meeting narrative, (3) Service co-production narrative and (4) Effective service process narrative. Based on the context, input, process and product (CIPP) model, they construct a plot narrative about the customer's need for individual guidance and the goal of transferring responsibility to the organisation. They create cross-pressure on the service process of lifestyle guidance. Achieving the effectiveness of lifestyle guidance requires the service provider to ensure the service is implemented more flexibly between general, self-directed and individually supportive guidance.

## Introduction

Effectiveness is key to a successful public service,^1,2^ that is to improving a customer's situation, which is achieved by a service or intervention in accordance with predetermined goals.^3^ As a concept, ‘effectiveness’ is ambiguous and difficult to define, as actors may pursue varied targets in the service process. Previous studies on effectiveness have focused on the outcomes, availability and costs of services in the medicine or health sectors,^3‐8^ without reconciling the views and experiences of the actors establishing service process effectiveness. However, lifestyle interventions have had positive effects on customers’ physical, mental, social and behavioural factors, especially weight management, physical and mental health, and physical activity.^4,7,9^ Lifestyle guidance can be seen as a motivator for the customer to change their health behaviour.^4,10^

In Finland, lifestyle guidance functions are based on the Current Care Recommendation, and the service's menu of methods is extensive. In this study, the working methods of weight management lifestyle guidance can be divided into four tools: information shown by measurement results, information production, discussion and functional methods.

A service's effectiveness is monitored with quantitative and qualitative indicators. The first and last times, the customer is measured for weight, waist circumference and certain blood counts. After each appointment, the employee uses structured recording to document the main content of the meeting in the patient records system. Besides discussions, such as motivational interviewing and reflective listening, information is produced via written instructions. The service emphasises the customer's active role, ie executive functions, and the process is designed to the customer's needs. The customer is not given ready-made answers but is expected to be responsible for their process: set goals, choose the tools that suit them best and evaluate the result. Therefore, in designing and implementing core services, coproduction is considered ‘a situation in which citizens are directly involved in producing [the] core services of an organisation and directly involved in both the design and implementation of the individual service provided to them’.^11^ The process takes about a year and covers six to eight meetings that are each one hour long. Meetings can be arranged in an individual or group format, in person or remotely.

In this study, lifestyle guidance effectiveness is examined using the CIPP (context, input, process and product) model, developed by Stufflebeam.^12^ When examining service effectiveness, the model's aims to determine the extent to which the service content meets customer needs.^13^ Context refers to needs, problems, resources and opportunities at different levels, which determine service process priorities. The purpose of input evaluation is to create a programme plan that meets customer needs^14^: examine the implementation of the selected approaches and methods in the everyday practices of the service process, according to the goals set in the strategy and action plan.^13^ The evaluation identifies the desired and possible results of the process.^13^ Concerning lifestyle guidance effectiveness, incorporating the views of several parties is essential,^4^ a consideration made when designing the research question: How is the construction of the effectiveness of the lifestyle guidance service process and the related customer orientation manifested in the narratives of the interface between customer experiences and the actors in the organisation within the framework of the CIPP model? The study aimed to provide descriptive knowledge of the service process based on coproduction from the perspective of customer orientation. Knowledge and understanding are built by combining the views of different actors.

## Method

The study focused on the lifestyle guidance process of the medium-sized Finnish hospital district of social welfare and health care. The hospital district was targeted because its region ranks among the top nationally for overweight and obese, with more than a quarter of women being overweight.^15^ The target organisation was granted a research permit and a decision that there was no need to review the research plan in an ethics committee (EKS/1678/13.01.05/2022). During the data collection (summer 2022), four lifestyle guidance customers, two employees and three managers were interviewed.

With the help of the thematic interviews, it was possible to focus on themes and questions from the perspective of the effectiveness of the lifestyle guidance process using the CIPP model.^13^ The interview themes dealt with the service process goals set by the representatives of the organisation, as well as the customers’ personal goals, the methods’ applicability and the process's ability to achieve effectiveness.

By interview time, the customers had been in the lifestyle guidance process for at least a year. They used individual services and group lifestyle guidance. Three customers were women and one male, aged 48 to 73 years, and three were working, and one retired. The interviews lasted from 50 to 90 min, with 57 pages transcribed from customer interviews and 79 from management and employee interviews.

The analysis of the data utilised a narrative thematic analysis method, which aimed to preserve the content of stories by theming them in a narrative manner.^16,17^ The analysis aimed to understand the subjective experiences and interpretations of the interviewees’ life guidance process, and goals, as well as those of the organisational representatives. The initial narrative themes began to emerge from the interviews after several readings, and they were related to expectations, need for support, suitable support and process strengths and challenges. Four main narratives were formed from the data, which are described with the type stories connecting representatives of the organisation and counter-stories formed from customers.

## Results

Based on the CIPP model, the four main narratives of the research data (Table 1) construct a plot narrative about the customer's need for individual guidance and the goal of transferring responsibility to the organisation. They create cross-pressure on the service process of lifestyle guidance. Cross-pressure brings together the contradictions of the main narratives, which describe the creation of effectiveness and customer orientation at the interface between actors.

**Table 1.** Four Main Narratives on the Formation of Cross-Pressure Between Effectiveness and Customer Orientation at the Interface Between Actors.

Main narratives of impact and customer orientation		Key question	CIPP model	Customer story	Conflicts between organisational and customer narratives	The cross-pressure between impact and customer orientation at the interface
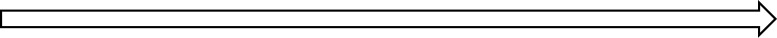						
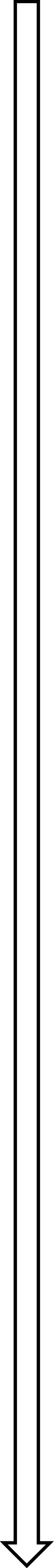	"The narrative of creating shared value”	To what extent does the service process meet customer needs?	Context	Ursula	Mismatch between the organisation's general lifestyle guidance and the customer's individual need for guidance	The need for individual guidance and the objective of delegating responsibility to the organisation
	"The narrative of meeting the organisation's strategy and customer needs”	To what extent does the organisation's strategy support a successful service process, ie, responding to customer needs?	Input	Hilja	Reconciling the scarcity of resources available to the organisation and general health goals with the individual service guidance needs of customers causes powerlessness	
	"The narrative of co-producing services”	What kind of strategies and service processes can be used to produce effective services?	Process	Martta	Utilising the resources and expertise of different services providers and combining them with the customer's need for individual guidance	
	"The narrative of an effective service process”	What kind of service process can be used to achieve results that support effectiveness	Product/ results	Aimo	The organisation's strong effort to produce impact despite the difficulties of measuring and utilising customer participation through successful individual guidance	

The narrative of an effective service process shows the organisation's strong effort to produce impact, despite the difficulties of measuring and utilising customer participation through successful individual guidance. However, measuring needs and goals promoted the efficiency of service targeting when the starting level of the customer's need for individual lifestyle guidance was defined and utilised by employees in service implementation. It also enables cost-effectiveness, as well as the implementation of preventive service goals in line with the organisation's and managerial strategy. The strategy narrative emphasises enabling participation according to employees and managers, which, from customer's perspective, requires concrete support for individual service needs. For two customers, the service was successful, because the methods of the service process were utilised in executive functions according to individual needs and resources. Concrete instructions and clear plans and their successful transfer into everyday life are crucial. The narrative of co-producing highlights the entire lifestyle guidance process, which was built complementarily by experts from different fields according to employees. Based on the narrative of creating shared value, two customers’ abilities were not sufficient to guide their activities to meet the organisation's general lifestyle guidance, which meant that the goals of the service process were not achieved. The research results show that effectiveness arose from the dynamics between the organisation's service production and the wishes and needs of customers.

## Discussion

According to previous studies, the effectiveness of lifestyle guidance services is increased by the planned lifestyle programme,^18^ mutual trust between service provider and customer, a consistent way of working,^19^ stakeholder support,^2^ genuine interest, understanding of disease risk factors,^18^ concretely verified measurements and set goals.^5,18^ It has also been estimated that the highest effectiveness in terms of guidance methods is achieved by combining different methods, such as close contact and remote meetings.^4,7^ The results of this study thus emphasise individual customer considerations in choosing the methods of lifestyle guidance services (eg co-produced by experts from different fields) to achieve effectiveness.

When building an effective service process, the organisation requires professionals and service organisations to develop functional methods that identify and respond to different and varying customer needs. This changes the service's customer orientation, where the role of the professional becomes situation-specific^18^ and considers individual changes^10^ and where the forms of service can be changed to achieve the desired impact.

## Limitations

Generalisation of the research results is limited due to the small sample size of qualitative research. By applying the CIPP model, the results can be examined more generally; thus, their transferability to other research on lifestyle guidance processes can be considered. A longitudinal study could have deepened the research data and provided better opportunities for more sustainable data-based interpretations. In addition to expanding the sample size, the diversity of participants should be considered in subsequent studies.

## Conclusion

Based on the study's findings, achieving the effectiveness of lifestyle guidance requires the service provider's ability to ensure flexibility in the way the service is implemented. The formation of a customer-oriented service, according to the customer's needs, is manifested in the transfer of responsibility between general, self-directed and individualistic guidance. In further research, the key questions are how organizational manage and employees can move from an organisation-centric to a customer-oriented approach and how the role of customers could be maximised to build genuinely effective services. Customer orientation in services emphasises that individual services are designed according to customer needs and ability to take responsibility, which requires sufficient organisational resources for planning and implementing customer guidance.
